# Comparative Assessment of In Vitro and In Silico Methods for Aerodynamic Characterization of Powders for Inhalation

**DOI:** 10.3390/pharmaceutics13111831

**Published:** 2021-11-02

**Authors:** Jelisaveta Ignjatović, Tijana Šušteršič, Aleksandar Bodić, Sandra Cvijić, Jelena Đuriš, Alessandra Rossi, Vladimir Dobričić, Svetlana Ibrić, Nenad Filipović

**Affiliations:** 1 Department of Pharmaceutical Technology and Cosmetology, University of Belgrade-Faculty of Pharmacy, Vojvode Stepe 450, 11221 Belgrade, Serbia; jelisaveta.ignjatovic@pharmacy.bg.ac.rs (J.I.); jelena.djuris@pharmacy.bg.ac.rs (J.Đ.); ibric@pharmacy.bg.ac.rs (S.I.); 2 Faculty of Engineering, University of Kragujevac, Sestre Janjić 6, 34000 Kragujevac, Serbia; tijanas@kg.ac.rs (T.Š.); aleksandarbodic.997@gmail.com (A.B.); 3 Bioengineering Research and Development Center (BioIRC), Prvoslava Stojanovića 6, 34000 Kragujevac, Serbia; 4 Department of Food and Drug, University of Parma, Viale delle Scienze 27/A, 43124 Parma, Italy; alessandra.rossi@unipr.it; 5 Department of Pharmaceutical Chemistry, Faculty of Pharmacy, University of Belgrade, Vojvode Stepe 450, 11221 Belgrade, Serbia; vladimir.dobricic@pharmacy.bg.ac.rs

**Keywords:** dry powders for inhalation (DPIs), computational fluid dynamics (CFD), discrete phase modeling (DPM), aerodynamic performance, solid lipid microparticles

## Abstract

In vitro assessment of dry powders for inhalation (DPIs) aerodynamic performance is an inevitable test in DPI development. However, contemporary trends in drug development also implicate the use of in silico methods, e.g., computational fluid dynamics (CFD) coupled with discrete phase modeling (DPM). The aim of this study was to compare the designed CFD-DPM outcomes with the results of three in vitro methods for aerodynamic assessment of solid lipid microparticle DPIs. The model was able to simulate particle-to-wall sticking and estimate fractions of particles that stick or bounce off the inhaler’s wall; however, we observed notable differences between the in silico and in vitro results. The predicted emitted fractions (EFs) were comparable to the in vitro determined EFs, whereas the predicted fine particle fractions (FPFs) were generally lower than the corresponding in vitro values. In addition, CFD-DPM predicted higher mass median aerodynamic diameter (MMAD) in comparison to the in vitro values. The outcomes of different in vitro methods also diverged, implying that these methods are not interchangeable. Overall, our results support the utility of CFD-DPM in the DPI development, but highlight the need for additional improvements in these models to capture all the key processes influencing aerodynamic performance of specific DPIs.

## 1. Introduction

Pulmonary drug delivery, as an alternative drug administration route, gained increased interest over the past few years. Among the various types of inhalation drug delivery devices, dry powder inhalers (DPIs) have been recognized for their benefits over, e.g., most commonly used metered dose inhalers (MDIs): (i) They do not include propellants; (ii) there is no need for coordination between device actuation and patient’s inhalation; (iii) they have improved stability; (iv) and they have better patient compliance [1,2,3]. While currently marketed DPIs include solely immediate-release products, recent research efforts have also been directed towards the development of sustained-release DPI formulations in order to reduce dosing frequency, and consequently increase patient compliance [4,5,6]. One of the approaches to sustain drug release from a DPI is based on the development of solid lipid microparticles (SLMs). In addition to controlled drug release, these formulations possess adequate aerodynamic properties [7]. The development of inhalable SLM powders has been described in literature [7,8,9,10,11,12,13,14,15,16,17,18,19,20]; however, the methods/apparatuses for their aerodynamic assessment, as the most important in vitro test, have been rather diverse, e.g., multistage liquid impinger (MSLI) [7,8,9,10,11,13,15,18], Andersen cascade impactor (ACI) [8,9,12], twin stage impinger (TSI) [19], fast screening impactor (FSI) [20] and next generation impactor (NGI) [20].

According to the European Pharmacopeia [21], all of the mentioned in vitro methods, except FSI, are acceptable for determination of DPI aerodynamic properties. On the other hand, abbreviated impactor measurements, such as FSI, are considered to be an easier and faster alternative for routine quality control purposes in comparison to the pharmacopeial tests [22,23]. In vitro assessment of a DPI aerodynamic performance is an inevitable test in DPI formulation development and a prerequisite to determine bioequivalence of a generic inhalation product [24,25]. Therefore, the availability of different in vitro methods can impede regulatory evaluation. Some studies compared two or three in vitro methods for aerodynamic assessment of DPIs [23,26,27,28,29,30,31,32,33], but their results were inconclusive. While some of the studies indicated similarities between ACI and NGI results [26,29,31], there were also reports on diverging results obtained by these two methods [28,29]. In addition, some differences were shown between NGI and MSLI [28], TSI [33] or FSI results [27].

Beside in vitro testing, contemporary trends of computer-aided drug development implicate the use of novel in silico methods to assess DPI aerodynamic properties. According to Wong et al., in silico computational fluid dynamics (CFD) modeling can be an effective tool in the design and optimization of DPI devices [34].

CFD models can simulate laminar and turbulent airflow and they are linked to fluid-particle dynamics models such as the discrete phase model (DPM), two-fluid model, mixture model, dense dispersed phase model and the discrete element method (DEM) to simulate aerosol particle flow and their interactions [35]. CFD can provide some of the key data related to DPI performance such as inhalation flow stream, particle trajectories, as well as data on particle detachment through flow stresses and impacts on the wall of the device. In the previous studies, the application of CFD in the assessment of DPI performance was mostly related to the analysis of the flow pattern and particle motion within the inhaler. The dependence of the inhaler’s performance on its geometry was also investigated in several studies [36,37,38]. These studies showed that parts of the inhaler such as mouthpiece, grid and air inlet size significantly affect the performance of the device. To exemplify this, the influence of airflow on DPI aerosolization was investigated in a study of Coates et al. [39], and revealed the best DPI dispersion performance at a flow rate of cc. 60 L/min. The authors applied different turbulence models for Turbuhaler^®^ DPI and compared the results with large eddy simulations (LES) and experimental data [40] to conclude that the k-ω SST turbulence model provided the most realistic results. Another study of Donovan et al. [41] assessed the influence of two different types of DPIs and changes in the physical properties (size and shape) of the carrier particles on aerosol performance, and found out that increase in the carrier particle diameter significantly increases the wall-impact rate in both types of DPIs, causing the cluster (carrier particles coated with drug powder) to disintegrate. A procedure describing the powder dispersion process using coupled CFD and DEM techniques has been described in the study of Tong et al. [42]. They showed that the dominant dispersion mechanism in the Aerolizer^®^ inhaler is agglomerate-wall impact in the region of the inhaler grid which results in increased fine particle fraction (FPF). To our knowledge, no published studies have examined particles’ behaviour after their impact with the wall in terms of whether the particles slide, roll or stick to the wall. However, there are studies such as the one by Milenkovic et al. [43] who investigated and defined the equations describing particles sticking to the wall, although no previous research has defined the mechanisms of particle flow after collision with the wall.

The review of Wong et al. (2012) emphasized that CFD simulation outcomes should always be validated by experimental results [34]. However, intrinsic differences between various in vitro, and in vitro and in silico methods complicate direct comparison between experimental and simulated data. A number of studies in parallel assessed the results of CFD simulations and in vitro experimental results (usually including a single in vitro method such as MSLI, NGI or ACI) [35,36,37,38,39,41,44,45,46,47,48,49,50,51,52,53]. Overall, some correlation between in vitro and in silico CFD results has been detected [48,49,50,51,52,53], but there is a need for additional comparative assessment of CFD tools in relation to the in vitro methods to evaluate the applicability of CFD in the research and development of various DPI formulations. A recent review study of Zheng et al. (2021) also concluded that more direct comparison between the simulated and experimental results is necessary to improve the prediction accuracy of in silico tools [54].

The aim of this study was to compare the designed CFD-DPM model outcomes with TSI, FSI and NGI results on aerosolization performance of SLM DPIs. CFD-DPM analysis was performed to describe the steady airflow and deposition processes of SLM DPIs within the RS01^®^ as a model DPI device, taking into account the behaviour of particles after impact with the wall, i.e., whether the particles will stick or bounce off the wall, as well as the mechanism of their detachment from the wall. This was achieved by writing user defined functions (UDFs) for particle tracking. To our knowledge, there are no published reports on such an approach, so this study will improve the current understanding of DPI performance and the underlying mechanisms, which is difficult to attain by relying solely on the in vitro experiments.

## 2. Materials and Methods

### 2.1. Materials

SLM DPI formulations, described in Ignjatović et al. [20], were used as test formulations in this study. SLM DPIs were composed of glyceryl dibehenate (Compritol^®^ ATO 888, Gattefossé, Lyon, France), poloxamer 188 (Kolliphor^®^ P188, BASF, Ludwigshafen, Germany), water and salbutamol-sulphate (SS) (Galenika, Belgrade, Serbia), and some of the prepared SLM formulations included trehalose (TCI Chemicals, Tokyo, Japan). Composition of the tested formulations is given in Table 1. Diammonium hydrogen phosphate purchased from J.T Baker (Deventer, The Netherlands) and phosphoric acid (85%) supplied from Merck (Darmstadt, Germany) were used for the preparation of phosphate buffer (pH 2.8). Hydroxypropylmethylcellulose (HPMC) size 3 capsules were obtained from Lonza Capsule Delivery Solutions (Capsugel^®^ Vcaps^®^ Plus DPI, Colmar, France) and RS01^®^ Dry Powder Inhaler device (high resistance, flow rate 60 L/min) was gifted by Plastiape^®^ S.p.a. (Osnago (LC), Italy). All other reagents, purchased from commercial suppliers, were of analytical grade. Fresh ultrapure water was supplied from a TKA water purification system (Niederelbert, Germany). A full list of abbreviations used in this study is provided in the Abbreviations section.

### 2.2. Methods

#### 2.2.1. SLM DPIs Micromeritic Properties

Five SLM DPI formulations, described in the study of Ignjatović et al. [20] were evaluated in this study. The selected formulations (Table 1) were chosen based on the results of aerodynamic assessment, presented in our previously published study [20]. These formulations were prepared by a melt-emulsification process in conjunction with spray-drying by varying the selected process parameters and formulation composition, as summarized in Table 1.

Micromeritic properties of the selected SLM DPIs have been explained in detail in a previously published study [20]. Table 2 solely states the most important micromeritic properties of the selected SLM formulations that were used as input parameters for CFD-DPM simulations. These properties include geometric particle size (expressed as cumulative undersize volume diameter at 10%, 50% and 90% of particle population: d_v10_, d_v50_ and d_v90_, respectively), particle size distribution (expressed as span values) and DPI true density. Geometric particle size distribution was obtained by the laser diffraction method, and DPI true density was determined by helium pycnometer [20].

#### 2.2.2. In Vitro Aerodynamic Assessment of SLM DPIs

Aerodynamic properties of the SLM DPIs were assessed by means of FSI, NGI and TSI, as described in the following subsections.

##### Fast Screening Impactor

The aerodynamic performance of SLM DPIs, assessed by FSI analysis, was already reported in our previous study [20]. In brief, an accurately weighed amount of SLM DPI was introduced into the HPMC capsule, which was then inserted into the RS01^®^ DPI device and pierced. The device was connected to the FSI (Copley Scientific, Nottingham, UK) and passed by the air stream for 4 s at 60 L/min. The glass filter of FSI was weighed before and after the air actuation in order to determine the amount of powder deposited on the filter (particles smaller than 5 µm), which represents the fine particle dose (FPD). In addition, the emitted dose (ED) was calculated as the amount of powder that left the DPI device and entered the FSI. FPF was calculated as the ratio between FPD and ED in percent, and the emitted fraction (EF) was calculated as the ratio between ED and metered dose (MD). MD here represents the total mass of the powder filled in HPMC capsules.

##### Next Generation Impactor

The aerosol performance of SLM DPIs was also assessed by NGI, and the procedure is explained in our previous study [20]. In brief, an accurately weighed amount of SLM DPI was introduced into the HPMC capsule, which was then inserted into the RS01^®^ DPI device and pierced. The device was connected to the NGI (Copley Scientific, Nottingham, UK) through the USP induction port, and passed by the air stream for 4 s at 60 L/min. After actuations of three capsules for each formulation, the amount of powder deposited in all components of the assembled NGI (induction port (IP), cups (stages S1—S7) and micro-orifice collector (MOC)), device and mouthpiece adapter was recovered and analysed by high performance liquid chromatography (HPLC) [20]. In the NGI analysis, MD represents the mass of drug quantified by HPLC, calculated by summing the drug recovered from the DPI device and the impactor (induction port, stages S1 to S7 and MOC). The ED was the amount of drug leaving the DPI device and entering the NGI (induction port, stages S1 to S7 and MOC). The mass median aerodynamic diameter (MMAD) was determined by plotting the cumulative mass percentage less than the stated aerodynamic diameter for each NGI stage on a probability scale versus the aerodynamic diameter of the stage on a logarithmic scale. The FPD is the mass of drug particles sized < 5 μm, which was calculated from the log-probability plot equation. The FPF and EF were calculated as described in the FSI study. Geometric standard deviation (GSD) was calculated according to Equation (1):

(1) $$\mathrm{GSD}=\sqrt{\frac{d_{84.16}}{d_{16.84}}}$$

where d_84.16_ and d_16.84_ represent the diameters of the cumulative aerosol mass at 84.16% and 16.84%.

##### Twin Stage Impinger

TSI analysis was conducted as a third in vitro method to evaluate SLM DPI aerodynamic performance. A TSI glass apparatus (Ph. Eur. 10.0, [21]) was properly assembled; then a water:methanol mixture in the ratio 50:50 (%, *v*/*v*) was introduced to stage 1 (7 mL) and stage 2 (30 mL), and the glass apparatus was connected to the rotary vane vacuum pump (RV 53, IN-ECO, Ružomberok, Slovak Republic). The airflow rate was set to 60 L/min using the flowmeter (R2, IN-ECO, Ružomberok, Slovak Republic), connected to the vacuum pump and glass apparatus. The RS01^®^ DPI device was filled with a size 3 HPMC capsule loaded with 15 mg of SLM DPI. The device was then connected to the TSI glass apparatus through the mouthpiece adapter. The HMPC capsule was pierced and the DPI device was activated and tested for 4 s at a flow rate of 60 L/min. After consequent actuations of three capsules, the glass apparatus was reassembled, then stages 1 and 2 were sonicated in the heated water bath (Bandelin, Sonorex RK 102H, Berlin, Germany) for 5 min and washed with water:methanol mixture into separate volumetric flasks. The DPI device and capsules were also separately washed into volumetric flasks. All the flasks were then sonicated for 5 min in order to melt the lipid matrix of the microparticles and dissolve the complete amount of SS. Samples were then filtered (0.45 µm, mixed cellulose esters, Carl Roth GmbH, Karlsruhe, Germany) and drug concentration was analysed by HPLC.

HPLC analysis was performed on a Dionex UltiMate 3000 system (Thermo Fisher Scientific, Germering, Germany) equipped with a quaternary pump, PDA detector (set to 276 nm) and an autosampler. The separation was performed using the Zorbax Eclipse XDB-C18 column (4.6 × 150 mm, 5 µm particle size, Agilent Technologies, Santa Clara, CA, USA), thermostated at 25 °C. The mobile phase, which consisted of phosphate buffer (pH 2.8) and methanol in the ratio 80:20 (%, *v*/*v*), was used at a flow rate of 1.0 mL/min. The phosphate buffer (pH 2.8) was prepared by dissolving 2.625 g diammonium hydrogen phosphate in 400 mL of ultrapure water, and phosphoric acid (85%) was used to adjust the pH value to 2.8. The sample injection volume was 20 μL. The method’s linearity (R^2^ = 1.0000) was confirmed over the concentration range 0.125–100 μg/mL, using standard aqueous solutions of SS. The sensitivity of the method was estimated in terms of limit of quantification (LOQ) and limit of detection (LOD). The determined LOQ and LOD were 0.125 μg/mL and 0.040 μg/mL, respectively. In addition, the instrument repeatability precision was also confirmed (RSD = 0.65%).

#### 2.2.3. CFD-DPM Modeling

**Figure 1:**
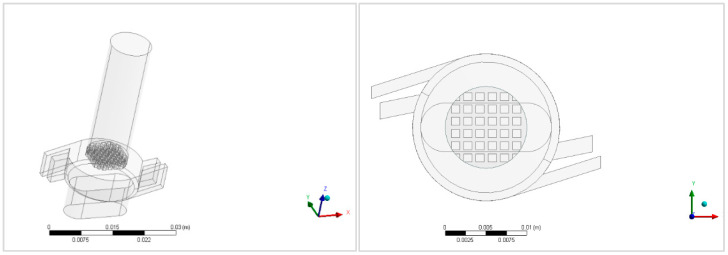
RS01^®^ inhaler’s geometry: Isometric view (**left**) and view above (**right**).

**Figure 2:**
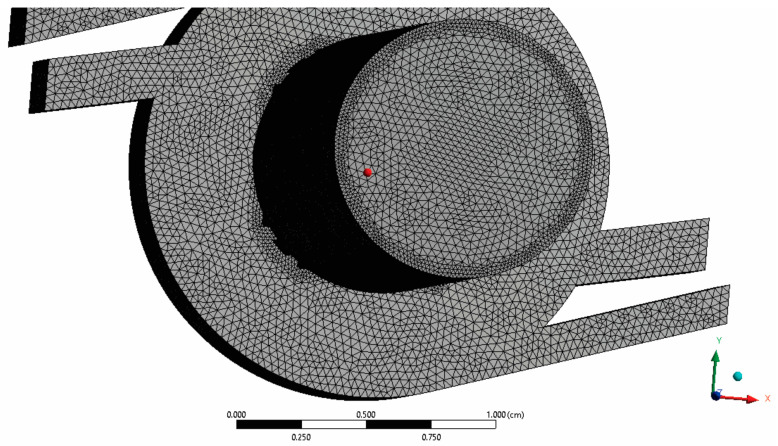
Finite volume mesh with near to wall refinement.

The RS01^®^ inhaler geometry (Figure 1) was created in a CAD/CAM environment (CATIA V5R20) [55] and then imported into the commercial software Ansys (version 16.0, ANSYS, Canonsburg, PA, USA) [56], where Ansys Meshing [57] was used to discretize the geometry into a finite volume mesh (Figure 2) with near to wall refinement, and Fluent (version 6.3, ANSYS, Canonsburg, PA, USA) [58] was used for numerical simulations.

The first step was to disassemble and measure the real physical DPI device and its component elements, such as height, width, diameter, etc. The measured dimensions of the RS01^®^ device were: (i) Maximal height: 47.5 mm; (ii) maximal width: 28.0 mm; (iii) outlet diameter: 10.0 mm; (iv) inlet dimensions: 1.5 mm × 6.0 mm.

The quality of the mesh was checked within Fluent Meshing, and then the mesh was used in Fluent to simulate the airflow. The edges and common planes were meshed initially, followed by the creation of various computational volume meshes. Refinements were made around corners, edges and planes corresponding to walls during the meshing phase. In areas where substantial velocity gradients were predicted, volumetric meshes were refined. It was concluded that at least seven grid points in the near wall region are required, y^+^ < 2.5. To meet this criterion, the computational grids in this study were improved (for Q = 60 L/min) at the near wall region. Computational meshes ranged from 2 × 10^5^ to 2 × 10^7^ and were made up of tetrahedral cells [59]. Maximum skewness of cells was 0.85. The total particle depositions for the six different meshes (approximately 2 × 10^5^, 5 × 10^5^, 1 × 10^6^, 2 × 10^6^, 5 × 10^6^ and 1 × 10^7^) were compared with 100% deposition assumed to determine the results’ independency based on mesh. Based on these simulations the 2 × 10^6^ mesh appeared to provide sufficient refinement to obtain accurate particle simulation results. In conclusion, the mesh with total number of nodes 349,460 and number of cells 1930,248 (≈2 × 10^6^) was found to provide essentially identical results as the 1 × 10^7^ mesh, and was therefore used to obtain the results presented in this study.

Boundary conditions included defining wall surfaces and inlet and outlet pressure. This study examined the airflow and particle deposition with a pressure drop which corresponds to the volumetric flow rate of 60 L/min. Fluid flow through the inhaler is a result of the difference between the inlet and outlet pressures. In order to achieve a volume flow of 60 L/min, as defined by the in vitro experimental setup (FSI, TSI and NGI) and based on the outlet surface area of the inhaler, it was calculated that the velocity at the inhaler outlet should reach the value of approximately 12 m/s. The defined velocity was achieved by setting a pressure drop to the value of 2500 Pa. To obtain the defined pressure drop, the pressure value at the inlet was set to 0 Pa, while the pressure at the outlet was set to −2500 Pa. The same logic for prescribing the difference of pressures between inlets and outlet was employed by Milenkovic at al. (2013) [40].

The Navier–Stokes equations for the fluid flow were solved using Fluent. Particle motion and deposition were described using a Eulerian-fluid/Lagrangian-particle approach. We did not investigate particle–particle interaction, but only particle–wall interaction. In general, particle–particle collisions can have a significant impact and must be considered. However, this interaction can be ignored if the particle volume fraction is smaller than around 10^−3^ [60]. The same conclusion has been given by Milenkovic (2015) who stated that the effect of particle–particle collisions and aggregation is insignificant after the initial powder dispersion [59]. As a result, because of the tiny volume fraction (10^−4^) in this work, the effect of particle–particle collisions was not examined nor taken into consideration. Powder dispersion was assumed to occur instantaneously based on the Rosin–Rammler distribution and release model.

The airflow through the inhaler was simulated using time-averaged conservation of mass and momentum equations, i.e., Navier–Stokes equations. These equations were linked with an adequate turbulent model in order to describe the turbulence. Navier–Stokes time-averaged equations are called Reynolds Averaged Navier–Stokes (RANS) equations and are defined by the following relation (Equation (2)):

(2) $$\frac{\partial {\bar{u}}_{i}}{\partial t}+\frac{\partial }{\partial x_{j}}\left({\bar{u}}_{j}{\bar{u}}_{i}\right)=-\frac{\partial \bar{p}}{\partial x_{i}}+\frac{\partial }{\partial x_{j}}\left[\upsilon \left(\frac{\partial {\bar{u}}_{i}}{\partial x_{j}}+\frac{\partial {\bar{u}}_{j}}{\partial x_{i}}-\bar{{u^{\prime }}_{i}{u^{\prime }}_{j}}\right)\right]$$

where the term $\bar{{u^{\prime }}_{i}{u^{\prime }}_{j}}$ represents the Reynolds stress tensor which depends only on the fluctuating velocities. Depending on the approach that defines Reynolds stresses, different RANS turbulent models have been developed. In this study, the shear stress transport (SST) *k* − *ω* turbulent model was used, which according to Milenković et al. [40] gives the most correct results in relation to the other turbulent models. The *k* − *ω* SST model combines *k* − *ω* and *k* − *ε* turbulent models to eliminate their disadvantages. It works by using *k* − *ω* in the inner part of the boundary layer and switching to *k* − *ε* in free-stream. According to this model, the turbulent kinematic viscosity (*ν_t_*), which defines the Reynolds stress tensor, is determined by calculating the turbulent kinetic energy (*k*) and specific turbulence dissipation rate (*ω*). Therefore, this turbulent model consists of two transport equations, one for k and the other for ω. The transport equation for turbulent kinetic energy is given by Equation (3):

(3) $$\rho \left(\frac{\partial k}{\partial t}+{\bar{u}}_{j}\frac{\partial k}{\partial x_{j}}\right)=\frac{\partial }{\partial x_{j}}\left[\left(\mu +\sigma^{*}\mu_{t}\right)\frac{\partial k}{\partial x_{j}}\right]+P_{k}-\beta_{k}\rho k\omega$$

The transport equation, Equation (4), for specific turbulence dissipation rate is given in the following form:

(4) $$\frac{\partial \omega }{\partial t}+U_{j}\frac{\partial \omega }{\partial x_{j}}=\alpha S^{2}-\beta \omega^{2}+\frac{\partial }{\partial x_{j}}\left[\left(\upsilon +\sigma_{\omega }\upsilon_{t}\right)\frac{\partial \omega }{\partial x_{j}}\right]+2\left(1-F_{1}\right)\sigma_{\omega 2}\frac{1}{\omega }\frac{\partial k}{\partial x_{i}}\frac{\partial \omega }{\partial x_{i}}$$

Turbulent kinematic viscosity is calculated using Equation (5):

(5) $$\upsilon_{t}=\frac{a_{1}k}{\max(a_{1}\omega ,SF_{2})}$$

where *a*_1_ is an empirically determined constant, *S* is defined by the strain rate tensor, and functions *F*_1_ and *F*_2_ determine the connection between these two models.

The particle sticking mechanism depends on a variety of parameters, including particle size, velocity, angle of impact and surface properties of the particle and the contact wall. It is usually the product of one or more of the following mechanisms: Van der Waals and electrostatic forces under dry conditions, and liquid bridge forces under wet conditions [61]. Van der Waals force arises from molecular interactions between two surfaces, in this case, between a particle and a wall. Electrostatic force contributes to the sticking process if the incoming particles are electrically charged in the gas or fluid stream.

The criteria for surface-sticking particles are defined by Dahneke [62]. He analysed the role of particle impact velocity on the rebound velocity of spherical shape particles. He stated that, as the normal impact velocity (*v_n_*) decreases, the significance of the sticking force increases, resulting in decreased rebound velocity. Under the critical value of the normal impact velocity, there is no rebounding of the particles and the particles stick to the surface. This velocity is referred to as the capture velocity. Using a mathematical model for the impact and adhesion of spherical particles, Brach and Dunn [63] calculated the capture velocity (*v_cr_*) based on the experimental data. The capture velocity is given by Equation (6):

(6) $$v_{cr}={\left[\frac{2E}{d_{p}}\right]}^{\frac{10}{7}}$$

where *E* is the El Batch parameter, defined in the paper of El-Din and Diab [64] and *d_p_* is a particle diameter. Parameter *E* is calculated based on Equation (7):

(7) $$E=0.51{\left[\frac{5\pi^{2}(k_{1}+k_{2})}{4\rho_{p}{}^{3/2}}\right]}^{\frac{2}{5}}$$

The terms *k*_1_ and *k*_2_ are defined by Equations (8) and (9):

(8) $$k_{1}=\left(\frac{1-v_{s}{}^{2}}{\pi E_{s}}\right)$$

(9) $$k_{2}=\left(\frac{1-v_{p}{}^{2}}{\pi E_{p}}\right)$$

*E_s_* and *E_p_* are the Young’s modulus values of the inhaler wall surface and particle materials, respectively, while *v_s_* and *v_p_* are Poisson’s ratio values for the inhaler wall surface and particle materials, respectively. A particle having normal impact velocity greater than the critical velocity *v_n_* > *v_cr_* will bounce in contact with the surface. This means that the particle deposition will happen if *v_n_* < *v_cr_*. All the other constants are defined in Table 3.

Deposited particles are released and re-suspended when the fluid forces are large enough to overcome the particle adhesion forces. Soltani and Ahmadi [66] investigated various particle detachment mechanisms. Particles can be detached by rolling and sliding, but rolling is the most likely detachment mechanism for spherical particles.

If the moment induced by the fluid forces at a certain stage on the particle–wall interaction interface is larger than the moment induced by the adhesion force, the particle begins to roll and hence is detached. This is described by Equation (10):

(10) $$F_{D}\left(\frac{d_{p}}{2}-b\right)+F_{L}a\geq F_{st}a$$

where *F_D_* is the drag force, *d_p_* is a particle diameter, *F_L_* is the lift force, *F_st_* is the adhesion force, *a* is the distance along the contact surface from the particle center (deformation of the particle along the surface) and *b* represents the deformation of the particle normal to the surface. Wang [67] investigated the effects of initial motion on a particle’s detachment from the surface and defined a condition for particle detachment by sliding. A particle will detach from the surface via this mechanism if the fluid drag force *F_D_* is strong enough to make the particle slide, i.e., when:

(11) $$F_{D}\geq k_{s}F_{st}$$

where *k_s_* is the coefficient of static friction between the particle and the wall.

After obtaining an initial solution, further set up was necessary (e.g., solution method, relaxation factor). At the end, convergence of numerical simulations was assumed in cases where the residuals were less than 10^−4^. After reaching a converged solution, the particles were instantaneously injected from the surface, also using steady flow. In this study, only steady-state airflow was assumed, which may be considered as a close approximation to dynamic inhalations once the flow rate reaches the peak inspiratory value. This justification was adopted based on the work of Milenkovic (2015) [59]. After the steady state solution for airflow was reached in Fluent, particles were introduced into the flow. In general, particles are considered to enter the simulation domain (are “injected”) at a given moment from a priori determined source lines or surfaces in CFD simulations [59]. All simulations in this study were run under the premise that powder release and dispersion occur quickly between the outlet of the powder storage cylinder and a virtual release surface located at a short distance (12 mm from the bottom of the powder storage) downstream. In this study, based on the assumptions adopted from Milenkovic (2015) [59], we also assumed that particles were injected from an injection surface with the velocity of the particles equal to the velocity of the fluid.

The model was tested with a different number of injected particles (100—40,000) in order to achieve a consistent solution despite the number of particles. Particle sizes, used as input parameters in the simulations, were taken from in vitro experiments (i.e., particle sizes were determined by laser diffraction method, as explained in our previously published study [20]). Namely, we used different geometric particle size (expressed as: d_v10_, d_v50_ and d_v90_) and particle size distributions (expressed as span values) for each of the five tested SLM formulations as inputs for CFD-DPM simulations (Table 2). To determine the limiting behavior, particle simulations were first run with a deposition efficiency of 100%. After setting up the boundary conditions, as well as simulation setup that provided the consistent results, we adopted the final simulations to be performed with 100 particles in order to reduce the computational time and resources which are rather demanding for this type of simulation. Particle sticking and detachment mechanisms were simulated by introducing UDF to define the boundary conditions at the wall of the device. The UDF was written to calculate the particle critical velocity using Equation (6), and comparison of this value with the normal velocity of the particle determines whether the particle sticks to the wall or bounces. Subsequently, the relation given by Equation (11) was applied to determine whether the detachment mechanism is sliding or rolling. Following the completed calculation, the UDF generated a file containing data on particles that stick, detach by rolling and detach by sliding.

The processing hardware included 8 GB of RAM and an Intel(R) Core (TM) i3-7020U CPU running at 2.30 GHz (4 CPUs), while computational time was 6–7 h. However, with the large number of particles involved, e.g., from 10–100 × 10^6^ particles, simulations would only be possible with the use of special hardware/software (meaning graphical user interface (GUI), CUDA, parallel processing etc.). This will be the focus of future research in order to reduce the computational time and increase the complexity of the modelled phenomena.

#### 2.2.4. Aerodynamic Particle Size Distribution of CFD-DPM Generated Aerosol Data

The results of CFD-DPM simulations for five SLM formulations, which include geometric particle diameter, density and mass of the particles leaving the inhaler, were used to calculate the corresponding MMAD and GSD values. The aerodynamic particle diameter (*d_ae_*) was calculated using Equation (12):

(12) $$d_{ae}=d_{g}\;\times \;\rho_{p}$$

where *d_g_* is a particle’s geometric diameter, and *ρ_p_* is the powder’s true density.

MMAD was determined by plotting the cumulative mass percentage vs. calculated cut-off aerodynamic diameter (*d_ae_*) on a logarithmic scale, based on linear interpolation between the nearest data points on either side of the cumulative 50th mass percentile value [68]. GSD was calculated using Equation (1). Such an approach has already been applied by Vulović et al. [35].

#### 2.2.5. Data Analysis

Correlation diagrams were constructed in order to compare the results (EF, MMAD, GSD and FPF) of the four tested methods (one in silico and three in vitro methods). The comparison was made based on the correlation coefficients (R^2^) between the plotted data in the correlation diagrams, and two additional parameters: Root mean square error (RMSE) and normalized root mean square error (NRMSE). The RMSE was calculated according to Equation (13) [69]:

(13) $$\mathrm{RMSE}=\sqrt{\frac{\sum_{i=1}^{N}{\left(P_{i}-O_{i}\right)}^{2}}{N}}$$

where *P_i_* represents predicted or test values, *O_i_* represents observed or reference values and *N* is the number of reference/test values.

The NRMSE was calculated using Equation (14) [69]:

(14) $$\mathrm{NRMSE}=\sqrt{\frac{\mathrm{RMSE}}{\bar{O}}}$$

where $\bar{O}$ is the mean of the observed (reference) values.

## 3. Results and Discussion

Computational modeling of a DPI device performance is a difficult challenge that involves modeling of airflow, powder dispersion, aggregate breakage and particle deposition. These are many phenomena that occur at various spatial and temporal scales and necessitate unique computational approaches. Particle collisions with the inhaler’s walls are mostly caused by inertial impaction. The rate of deposition is determined by the particle–wall collision frequency and capture efficiency (regulated by adhesion forces), whereas the rate of collision-induced breaking is determined by particle cohesion forces. The key outputs of the model are the emitted flow (EF), as well as total number of deposited particles (that remain in the inhaler) and size distribution of the emitted particles.

**Figure 3:**
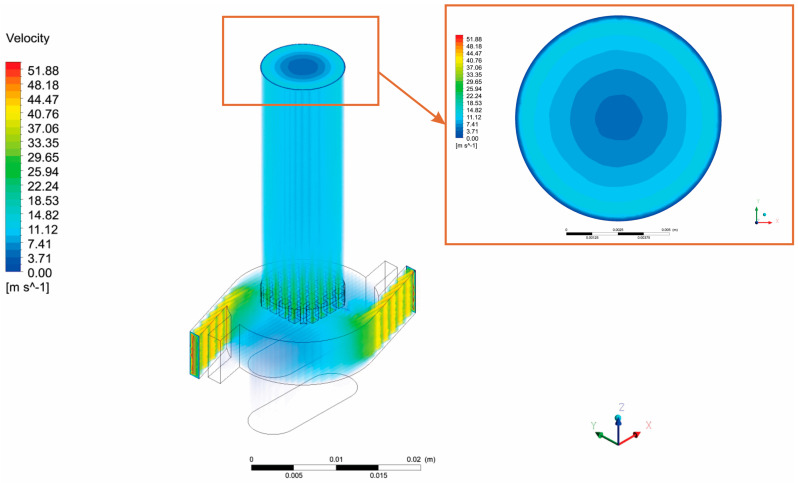
Velocity distribution inside the RS01^®^ DPI device with the outlet velocity distribution.

Initially, it is important to determine the fluid flow, in order to be able to track particles. The mouth outlet of the DPI device is critical because it determines particle dispersion and outflow from the DPI device to the oral cavity and upper respiratory tract. In this work, a volumetric airflow of Q = 60 L/min was applied. From the CFD simulation, a velocity distribution of the whole device, along with the outlet contour velocity distribution is shown in Figure 3.

**Figure 4:**
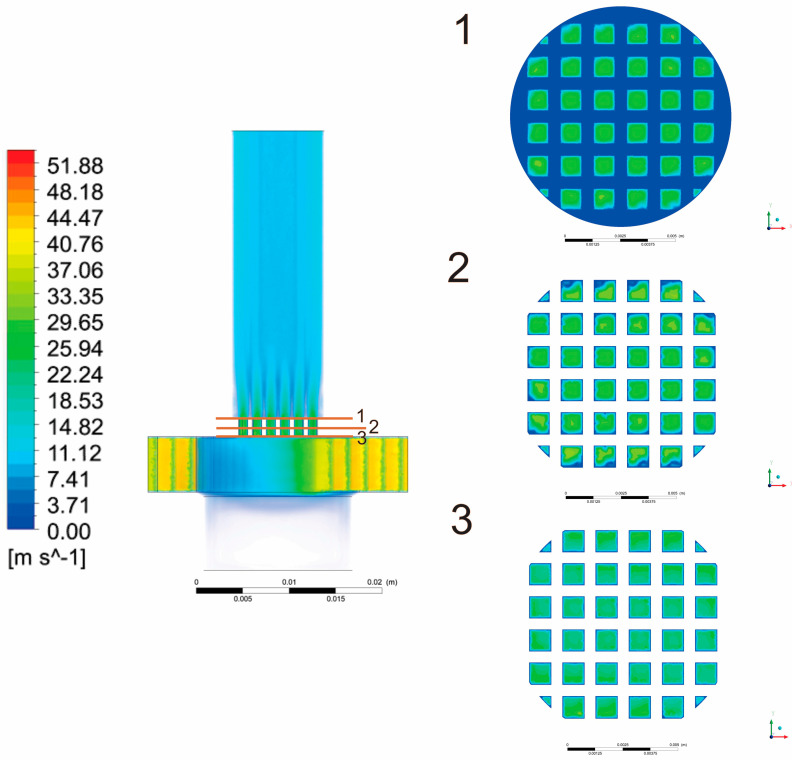
Velocity distribution inside the DPI device with velocity distribution at the characteristic cross sections.

The velocities in the middle part of the inhaler cause swirl flow with a tendency to carry the particles to the outlet. Beside the outlet, several cross sections of the inhaler are also of interest, such as the mesh grid section. These cross sections present the contour plots of the velocity magnitude on a plane parallel to the *z* axis. Velocity contours in these cross sections are shown in Figure 4.

After finding a steady state solution for the fluid, we introduced the particles to the model and set up the prescribed velocities for the particles based on the solution for the fluid. When the steady state solution was achieved, particle tracking was initialized with the defined UDF. First, we simulated dispersion of a larger number of particles to determine if the obtained particle impaction velocities were independent of the number of particles included in the model. Since the velocities proved to be independent of particles number, we decided to continue the simulations with a smaller number of particles, as simulation time and the required computer memory grow dramatically with an increase in particle number.

Simulations were performed for five formulations, F1–F5 (Table 1). Based on a literature review, and the fact that we could not determine material properties of the particles in our experiment, we adopted a Poisson coefficient for particles to be *v_p_* = 0.4 and Young’s module of elasticity to be *E_p_* = 1.0 Gpa, while for the inhaler wall material we used polystyrene surfaces (*v_s_* = 0.35 and *E_s_* = 4.1 GPa). The DPI wall equations, as defined in the Materials and Methods section (Section 2.2.3), were used to calculate the capture efficiency of particles.

**Figure 5:**
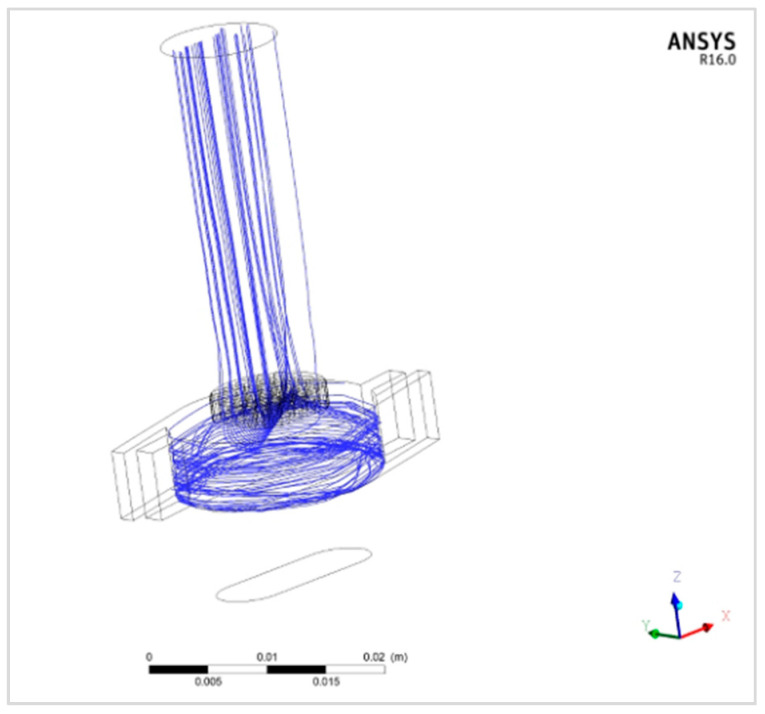
Particle trajectories for F1 particles.

The results show the particles’ trajectories inside the inhaler, and an example of particle trajectories for formulation F1 is depicted in Figure 5.

**Figure 6:**
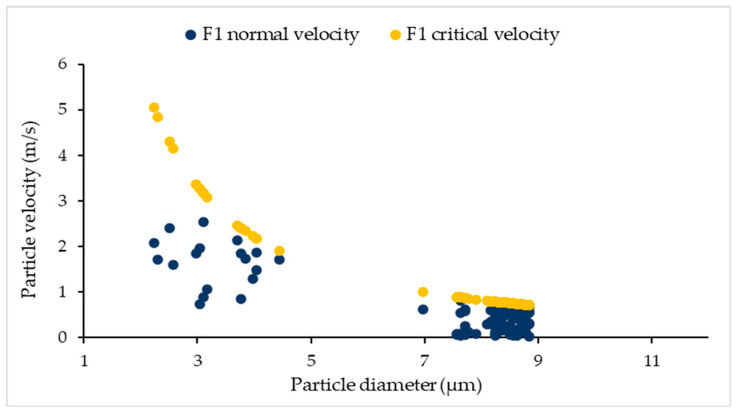
Normal and critical velocities for particles in formulation F1.

As already mentioned in the Materials and Methods section (Section 2.2.3), we tracked particles with their velocities in order to determine the mechanism of their behaviour. Particles with a normal impact velocity greater than the critical velocity *v_n_* > *v_cr_* will bounce upon contact with the inhaler wall surface. This means that particle sticking will happen if *v_n_* < *v_cr_*. Figure 6 shows normal and critical velocities for particles of different diameters in formulation F1 that have been stuck to the wall at some point. It can be seen that for particles with diameters in the 2–5 µm range, the difference between *v_n_* and *v_cr_* is larger than for particles with diameters in the 7–9 µm range, implying that the sticking mechanism is evident for smaller particles, whereas for larger particles this mechanism is more complex, as breakage may occur.

**Figure 7:**
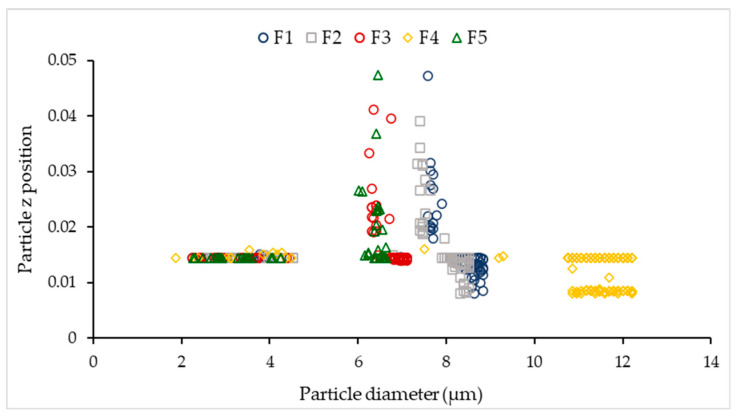
Relationship between the z position and diameter of particles in formulations F1–F5.

Analysis of the relationship between a particle z position and particle diameter for the deposited particles (Figure 7) indicates that smaller particles with a diameter in the 2–5 µm range will more likely stick only in the lower parts of the inhaler, whereas particles with a diameter in the 6–10 µm range may stick in both the lower and the upper parts of the inhaler. Particles with diameter 10–13 µm will again stick only in lower parts of the inhaler.

In order to further analyse CFD-DPM predictions and compare them with the in vitro results, we examined several parameters defining the aerodynamic performance of the model formulations.

**Figure 8:**
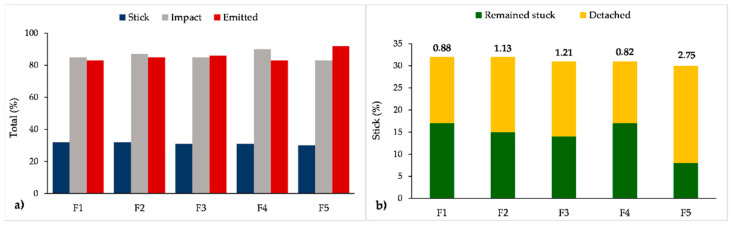

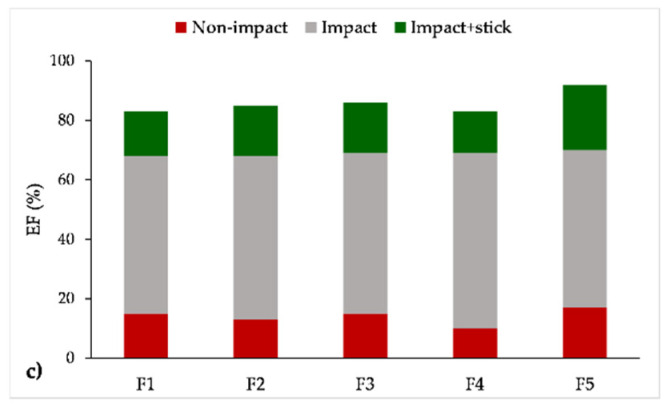
Total fraction of particles that stick to the wall, impact the wall and become emitted from the inhaler (EF—emitted fraction) (**a**); total fraction of particles that remain stuck or detach from the wall (the numbers above bars represent the ratio between percent of total particles that detach from the wall and percent of total particles that remain stuck to the wall (**b**); fraction of the emitted particles that impact, impact and stick or do not impact the wall (**c**).

Figure 8a shows CFD-DPM predictions on the fractions of particles that impact the inhaler wall, stick to the wall or leave the inhaler (EF). Considering all the formulations, between 83% and 90% of particles impacted the inhaler wall (Figure 8a). Some of them were stuck to the inhaler wall, and some left the inhaler. Approximately 30% of total particles stuck to the wall at some moment, but a different percentage of these particles subsequently detached from the wall (Figure 8b) and left the inhaler. This led to the differences in the EF between the formulations since the particles that remained stuck to the wall could not leave the inhaler. For this reason, the predicted EFs varied between 83% and 92% (Figure 8a,c). According to the simulations, the highest EF was estimated for formulation F5 (EF = 92%) which had the smallest percent of particles that impacted the wall (83%; Figure 8a) and the largest ratio between the percent of total particles that detached from the wall and the percent of total particles that remained stuck to the wall (Figure 8b). It was also observed that larger particles will more likely stick to the wall and remain in that position, while smaller particles that were stuck to the wall will detach more easily and eventually leave the inhaler. For example, formulation F5 had the smallest particle size and size distribution (Table 2), resulting in the lowest possibility for the particles to stick to the walls and remain in the inhaler, i.e., most of the particles that impacted the wall and stuck were eventually emitted from the inhaler (Figure 8c).

**Figure 9:**
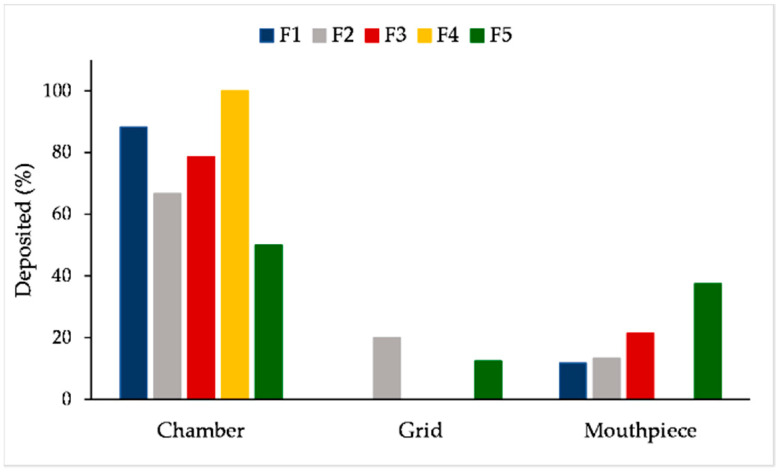
CFD-DPM predicted regional particle deposition in the RS01^®^ inhaler. CFD-DPM—computational fluid dynamics and discrete phase model.

**Figure 10:**
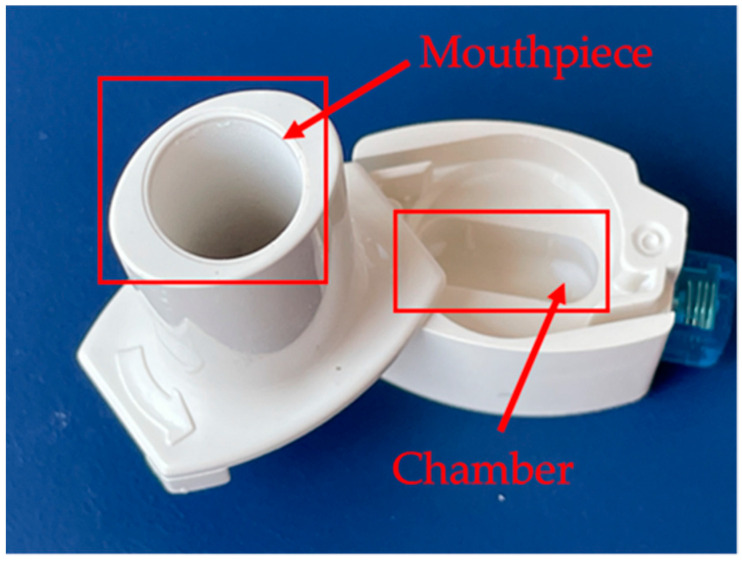
Particle deposition of F5 in the inhaler following RS01^®^ activation in the TSI study. TSI—twin stage impinger.

CFD-DPM outcomes also indicated the position of the particles that remained (deposited) in the inhaler, i.e., stuck to some part of the inhaler (chamber, grid or mouthpiece). The distribution of particles deposited in each of these regions is represented in Figure 9. In the case of all formulations, the largest percent of particles was estimated to deposit in the RS01^®^ chamber. However, a large fraction of particles that remained in the chamber belongs to the border size particles that may deposit either in the chamber or in the inhaler’s grid. The largest fraction of border size particles was estimated for formulation F4 (cc. 59%). In silico simulations revealed that smaller particles have a tendency to deposit in the inhaler’s chamber or between the chamber and grid, as well as the largest particles, while particles of diameter 6–10 µm may also deposit in the mouthpiece. It can be observed that particles from formulation F4, which had the largest size, did not deposit in the mouthpiece, while in the case of formulation F5, a considerable percent of particles deposited in the mouthpiece. The CFD-DPM outcomes, indicating that the largest fraction of SLM particles remain in the RS01^®^ chamber, coincide with the observations from the in vitro, e.g., TSI studies, illustrated in Figure 10.

**Figure 11:**
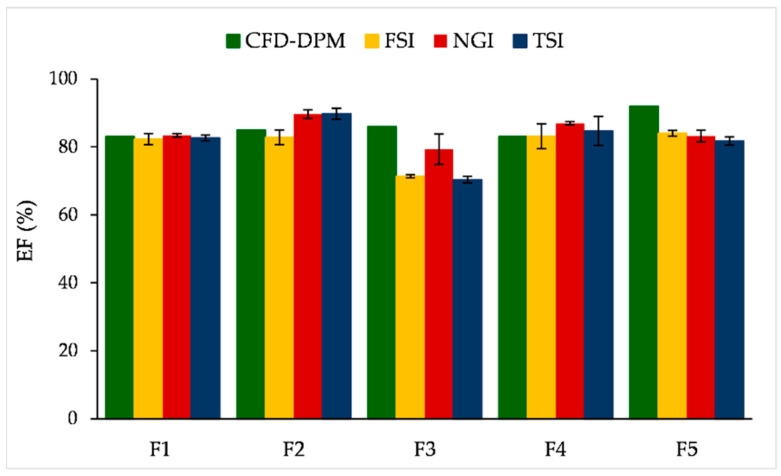
In vitro results (average values ± standard deviations) and CFD-DPM predictions for EFs of the tested SLM DPIs (FSI results were taken from Ignjatović et al. [20]). CFD-DPM—computational fluid dynamics and discrete phase model; DPI—dry powder for inhalation; EF—emitted fraction; FSI—fast screening impactor; NGI—next generation impactor; SLM—solid lipid microparticle; TSI—twin stage impinger.

In the following step, CFD-DPM predicted results for EFs have been compared with the results of different in vitro experimental methods (Figure 11).

It can be observed (Figure 11) that CFD-DPM simulated EF values are mostly comparable to the EF values obtained by the three in vitro methods. Namely, CFD-DPM predicted EFs matched well with the in vitro obtained EFs for three formulations (F1, F2 and F4), but bigger differences between the resulting EFs were observed in the case of F5 and especially F3 formulations. The highest CFD-DPM predicted EFs for formulations F3 and F5 were probably caused by the smallest geometric size distribution used as input in the CFD-DPM, i.e., as previously explained, according to the CFD-DPM simulations smaller particles have a lesser tendency to permanently stick to the inhaler’s wall, meaning they will more easily leave the inhaler. In contrast, the in vitro results indicated the lowest EF for formulation F3 which might be explained by possible trehalose induced agglomerate formation of relatively small SLM particles (both F3 and F4 contain trehalose, but F3 has smaller particles); however, this phenomenon could not be simulated by the designed CFD-DPM model. Due to these differences, no correlation could be established between CFD-DPM predictions and the results of three in vitro methods (Table 4). On the other hand, a relatively high correlation coefficient (0.8969) was observed for EFs determined by NGI and TSI, while the correlation between NGI and FSI results was much lower (Table 4). In addition, a certain level of correlation was noticed between TSI and FSI results (R^2^ = 0.7686), together with the lowest RMSE and NRMSE values (3.380 and 0.041, respectively), as represented in Table 4. These results implicate that both simple in vitro methods (FSI and TSI) could be used for DPI EF determination in a screening phase of DPI development, but TSI results correlate better with NGI measurements.

**Figure 12:**
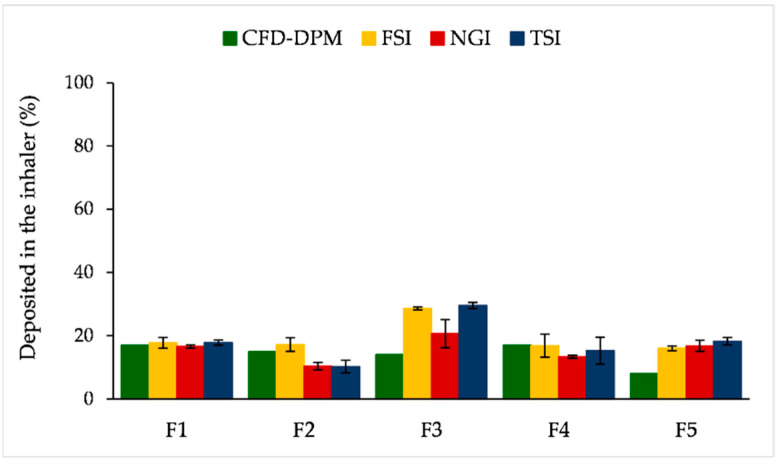
CFD-DPM and in vitro estimated SLM fractions deposited in the RS01^®^ inhaler. CFD-DPM—computational fluid dynamics and discrete phase model; FSI—fast screening impactor; NGI—next generation impactor; SLM—solid lipid microparticle; TSI—twin stage impinger.

Figure 12 illustrates percent of deposited particles in the RS01^®^ device, obtained by four different methods. Again, it can be observed that CFD-DPM predicted deposited fractions in the case of F1, F2 and F4 were rather similar to the deposited fractions obtained by FSI. Moreover, the largest difference between CFD-DPM and the in vitro results was noted for formulation F3. As discussed above, smaller CFD-DPM predicted fraction of the deposited particles for F3 might be caused by the inability of the CFD-DPM model to simulate agglomerate formations and their concomitant deposition in the inhaler.

**Figure 13:**
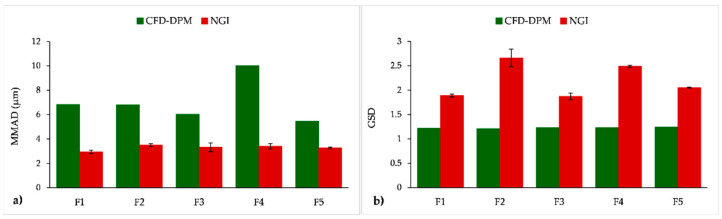
CFD-DPM and in vitro (NGI) estimated MMAD (**a**) and GSD (**b**) values for the tested formulations. CFD-DPM—computational fluid dynamics and discrete phase model; GSD—geometric standard deviation; MMAD—mass median aerodynamic diameter; NGI—next generation impactor.

**Figure 14:**
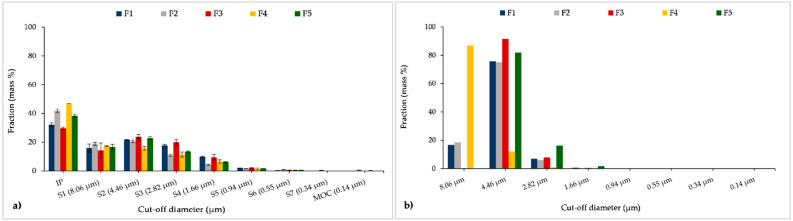
Fractional aerodynamic particle size distribution determined by NGI (i.e., deposited on different stages of NGI) (**a**) and predicted by CFD-DPM (**b**). CFD-DPM—computational fluid dynamics and discrete phase model; IP—induction port; MOC—micro-orifice collector; NGI—next generation impactor; S1 to S7—impactor stages 1 to 7.

We also analysed and compared CFD-DPM and in vitro estimated MMAD and GSD values, since these two parameters are important indicators of a DPI aerodynamic performance. Among the applied in vitro methods, only NGI results could be used to calculate MMAD and GSD; therefore, only NGI results were compared to CFD-DPM simulations (Figure 13). The obtained data demonstrate that CFD-DPM predicted MMAD values were almost twice as large as the corresponding values obtained by NGI. In addition, CFD-DPM predicted results indicated larger differences between MMAD for different formulations in comparison to the in vitro results showing similar MMAD for all tested formulations. According to the CFD-DPM outcomes, the largest MMAD was obtained for formulation F4 because the CFD-DPM predicted aerodynamic diameter was calculated based on true density and geometric particle diameter, and both of these parameters were the highest for particles in formulation F4. However, other phenomena such as the influence of trehalose on the aerodynamic performance of SLM DPIs were not simulated by the CFD-DPM model, which might be considered as a drawback of the in silico method. In contrast to MMAD results, CFD-DPM predicted GSD values were the same for all formulations, and lower than NGI obtained values. These results can be explained by the differences in the CFD-DPM and NGI estimated aerodynamic particle size distribution (Figure 14). To enable direct comparison between CFD-DPM and NGI results, the cut-off values for particle diameters (Figure 14) were set to comply with cut-off values for different NGI stages. It can be observed that CFD-DPM results indicated narrower aerodynamic particle size distribution than NGI, whereas the majority of particles in all formulations were distributed within two or three size groups (Figure 14b). Such results explain lower CFD-DPM predicted GSD values in comparison to NGI (Figure 13b). This resulted in a lack of correlation between MMAD and GSD results from CFD-DPM and NGI methods (Table 5).

**Figure 15:**
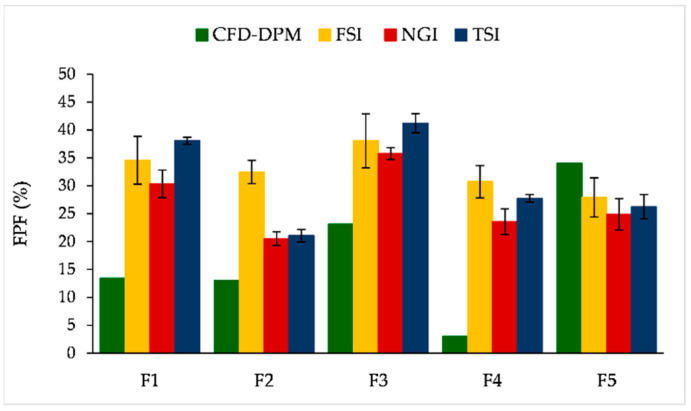
CFD-DPM predicted and in vitro obtained (FSI, NGI and TSI) FPF (%). CFD-DPM—computational fluid dynamics and discrete phase model; FPF—fine particle fraction; FSI—fast screening impactor; NGI—next generation impactor; SLM—solid lipid microparticle; TSI—twin stage impinger.

FPF is another parameter of interest analysed in this study. Figure 15 represents CFD-DPM and in vitro estimated FPF values. The results show that CFD-DPM predicted FPFs were notably lower than the in vitro obtained values for all formulations except F5 (Figure 15). A similar observation—that CFD-DPM predicted FPFs were lower in comparison to experimental values—was made in a study of Tong et al. (2011) [45]. In addition, there was no correlation between the in silico predicted and in vitro determined FPF values for the tested formulations (Table 6). The biggest difference between the in vitro and CFD-DPM results was again observed for formulation F4, and the underlying reason is explained above for MMAD values (here, a higher MMAD value complies with a lower FPF value).

Comparative analysis of the in vitro results revealed that TSI and FSI testing indicated higher FPF values than NGI results (Figure 15). The differences between TSI and NGI results are probably caused by the fact that TSI considers particles smaller than 6.4 µm to be respirable, whereas the cut-off aerodynamic diameter for NGI FPF values is 5 µm. Similar findings referring to the higher FPF values obtained by TSI in comparison to NGI, due to the difference in the cut-off value for FPF, were observed by Omer et al. [33]. Higher FPF values obtained by FSI in comparison to NGI have already been recognized as a limitation of FSI [27,70], although some studies showed no significant differences between FSI and NGI results, e.g., [23,30]. The highest level of correlation (R^2^ = 0.9440) with the lowest values of RMSE and NMRSE (4.669 and 0.173, respectively) was observed between FPF results from NGI and TSI, while the correlation between NGI and FSI, and TSI and FSI results was less pronounced (Table 6). In other words, the in vitro results on FPF values support our previous comment that TSI might be a convenient test for fast formulation screening in DPI development.

## 4. Conclusions

CFD modeling for various DPI devices (Aerolizer^®^, Turbuhaler^®^, Twincer^®^, Handihaler^®^, Accuhaler^®^, Multihaler^®^, etc.) has already been described [54], but to the best of our knowledge the CFD-DPM model of RS01^®^ inhaler has not been published in the available literature.

The CFD-DPM inhaler model designed in this study was improved in comparison to our previous model described in a study of Vulović et al. [35]. In the previous study [35], we used an idealised assumption that 100% of particles will reflect (bounce off) after impact with the wall, which is unrealistic, but greatly reduces the model complexity. In this study, we introduced equations to describe the particle-to-wall sticking mechanisms. Simulation results for the tested formulations revealed that particles will either stick to the wall or bounce off with reduced velocity, but no sliding and rolling would occur.

Considering a variety of parameters that can be predicted only by a CFD-DPM (e.g., regional particle deposition in the inhaler, fractions of particles that impact, stick, detach from the inhaler’s wall etc.), and the ability to mechanistically describe particle trajectories and interactions within the inhaler, in silico CFD-DPM modeling can be considered as a useful tool in DPIs development. In addition, CFD-DPM offers the ability for parallel screening of various aerodynamic parameters, e.g., this method can provide primary data on the fraction of drug/powder leaving the inhaler (EF (%)), and can (roughly) predict other important aerodynamic characteristics like FPF and MMAD (and GSD), while some simpler in vitro methods (such as FSI and TSI) can only determine FPF and EF, and for MMAD determination more laborious in vitro methods (such as NGI) are necessary. The in vitro results indicated that the outcomes of different in vitro methods are not comparable and that these methods are not interchangeable. Moreover, a satisfying level of correlation between NGI and TSI results indicates that TSI could be used as a fast screening method in DPIs development.

Overall, our results support the utility of CFD-DPM in DPIs development but highlight the need for additional improvements in the in silico models to capture all the key processes (e.g., particle (de)agglomeration) influencing the aerodynamic performance of specific DPI formulations such as SLMs.

## Figures and Tables

**Table 1:** Composition of the selected formulations and the applied process parameters ^1^.

Formulation	Lipid (%)	Poloxamer 188 (%)	SS (%)	Trehalose Addition	High Shear Mixing Time (min)	High Shear Mixing Speed (rpm)	T Inlet (°C)	T Outlet (°C)	Feed Rate (mL/min)	Spraying Airflow Rate (L/h)	Wash
F1	5.00	0.40	1.00	No	8.00	13,400	89	58	1.80	473	No
F2	5.00	1.50	1.00	No	2.00	13,400	89	58	1.80	670	No
F3	5.00	1.50	2.00	Yes	2.00	13,400	89	60	1.80	670	Yes
F4	5.00	1.50	1.00	Yes	2.00	13,400	89	56	1.80	670	No
F5	5.00	1.50	1.00	No	2.00	13,400	80	50	1.80	670	Yes

^1^ Taken from Ignjatović et al. [20]. SS—salbutamol-sulphate; T—temperature.

**Table 2:** SLM DPI micromeritic properties ^1^.

Formulation	d_v10_ (µm)	d_v50_ (µm)	d_v90_ (µm)	Span	True Density (g/cm^3^)
F1	2.24 ± 0.04	4.42 ± 0.07	8.84 ± 0.37	1.492 ± 0.071	1.050 ± 0.001
F2	2.43 ± 0.02	4.67 ± 0.03	8.51 ± 0.06	1.304 ± 0.003	1.060 ± 0.002
F3	2.20 ± 0.05	4.03 ±0.06	7.10 ± 0.03	1.217 ± 0.025	1.170 ± 0.001
F4	1.87 ± 0.01	5.13 ±0.09	12.21 ± 0.06	2.016 ± 0.034	1.230 ± 0.001
F5	2.26 ± 0.02	3.94 ± 0.03	6.63 ± 0.04	1.110 ± 0.003	1.030 ± 0.002

^1^ Taken from Ignjatović et al. [20]. DPI—dry powder for inhalation; d_v10_, d_v50_ and d_v90_—cumulative undersize volume diameter at 10%, 50% and 90% of particle population, respectively; SLM—solid lipid microparticle.

**Table 3:** List of constants used to define particle sticking behaviour.

Parameter Name	Symbol	Value	Unit	Reference
Young’s modulus for the inhaler wall surface	*E_s_*	4.1 × 10^9^	Pa	[43]
Young’s modulus for particle	*E_p_*	1 × 10^9^	Pa	[43]
Work of adhesion	*W_A_*	0.039	J/m^2^	[65,66]
Poisson’s ratio for the inhaler wall surface	*v_s_*	0.35	/	[43]
Poisson’s ratio for particle	*v_p_*	0.40	/	[43]
Particle density	*ρ_p_*	Taken from Table 2	kg/m^3^	/
Air density (at 1013.25 h Pa (abs) and 15 °C)	*ρ*	1.225	kg/m^3^	[43]
Dynamic viscosity of fluid	*µ*	1.7894 × 10^−5^	N s/m^2^	[43]
Correction factor for the near wall	*f*	1.70	/	[65,66]
Cunningham correction factor	*C_u_*	1 (for spherical particles)	/	[65,66]
Static coefficient of friction	*k_s_*	0.50	/	[65,66]

**Table 4:** Correlation coefficient (R^2^), root mean square error (RMSE) and normalized root mean square error (NRMSE) between EF (%) obtained by four different methods.

	NGI **			TSI **			FSI **		
	R^2^	RMSE	NRMSE	R^2^	RMSE	NRMSE	R^2^	RMSE	NRMSE
CFD-DPM *	0.0847	5.643	0.067	0.0342	8.651	0.106	0.0055	7.531	0.093
FSI *	0.5064	5.041	0.060	0.7686	3.380	0.041	/	/	/
TSI *	0.8969	4.183	0.049	/	/	/	/	/	/

* Represents the method used to obtain predicted or test values; ** Represents the method used to obtain observed or reference values. CFD-DPM—computational fluid dynamics and discrete phase model; EF—emitted fraction; FSI—fast screening impactor; NGI—next generation impactor; TSI—twin stage impinger.

**Table 5:** Correlation coefficient (R^2^), root mean square error (RMSE) and normalized root mean square error (NRMSE) between MMAD and GSD from CFD-DPM and NGI.

	R^2^	RMSE	NRMSE
MMAD	0.0643	4.044	1.232
GSD	0.2215	1.020	0.465

CFD-DPM—computational fluid dynamics and discrete phase model; GSD—geometric standard deviation; MMAD—mass median aerodynamic diameter; NGI—next generation impactor.

**Table 6:** Correlation coefficient (R^2^), root mean square error (RMSE) and normalized root mean square error (NRMSE) between FPF (%) obtained by four different methods.

	NGI **			TSI **			FSI **		
	R^2^	RMSE	NRMSE	R^2^	RMSE	NRMSE	R^2^	RMSE	NRMSE
CFD-DPM *	0.0789	14.178	0.525	0.0111	18.263	0.592	0.0219	19.227	0.587
FSI *	0.5948	6.723	0.249	0.5868	5.726	0.186	/	/	/
TSI *	0.9440	4.669	0.173	/	/	/	/	/	/

* Represents the method used to obtain predicted or test values; ** Represents the method used to obtain observed or reference values. CFD-DPM—computational fluid dynamics and discrete phase model; FPF—fine particle fraction; FSI—fast screening impactor; NGI—next generation impactor; SLM—solid lipid microparticle; TSI—twin stage impinger.

## Data Availability

Not applicable.

## Abbreviations

*a*	Distance along the contact surface from the particle center
*a*_1_	Empirically determined constant
ACI	Andersen cascade impactor
*b*	Deformation of the particle normal to the surface
CFD	Computational fluid dynamics
*Cu*	Cunningham correction factor
*d_ae_*	Aerodynamic diameter
*d_g_*	Geometric diameter
*d_p_*	Particle diameter
DPIs	Dry powders for inhalation and dry powder inhalers
DEM	Discrete element method
DPM	Discrete phase modeling and discrete phase model
*E*	El Batch parameter
ED	Emitted dose
EF	Emitted fraction
*E_s_*	Young’s modulus for the inhaler wall surface
*E_p_*	Young’s modulus for particle
*f*	Correction factor for the near wall
*F_D_*	Drag force
*F_L_*	Lift force
*F_st_*	Adhesion force
FPD	Fine particle dose
FPF	Fine particle fraction
FSI	Fast screening impactor
GSD	Geometric standard deviation
HPLC	High performance liquid chromatography
HPMC	Hydroxypropylmethylcellulose
IP	Induction port
*k*	Turbulent kinetic energy
*k_s_*	Static coefficient of friction
LES	Large eddy simulations
LOD	Limit of detection
LOQ	Limit of quantification
MD	Metered dose
MDIs	Metered dose inhalers
MMAD	Mass median aerodynamic diameter
MOC	Micro-orifice collector
MSLI	Multistage liquid impinger
*N*	Number of reference/test values
NGI	Next generation impactor
NRMSE	Normalized root mean square errror
$\bar{O}$	Mean of the observed (reference) values
*O_i_*	Observed or reference values
*P_i_*	Predicted or test values
R^2^	Correlation coefficient
RANS	Reynolds Averaged Navier Stokes
RMSE	Root mean square error
SLMs	Solid lipid microparticles
SS	Salbutamol-sulphate
SST	Shear stress transport
TSI	Twin stage impinger
UDFs	User defined functions
$\bar{{u^{\prime }}_{i}{u^{\prime }}_{j}}$	Reynolds stress tensor
*v_cr_*	Capture velocity
*v_n_*	Normal impact velocity
*v_p_*	Poisson’s ratio for particle
*v_s_*	Poisson’s ratio for the inhaler wall surface
*v_t_*	Turbulent kinematic viscosity
*W_A_*	Work of adhesion
*µ*	Dynamic viscosity of fluid
*ρ*	Air density
*ρ_p_*	Particle density
ω	Specific turbulence dissipation rate
